# Effects of Tasting and Ingredient Information Statement on Acceptability, Elicited Emotions, and Willingness to Purchase: A Case of Pita Chips Containing Edible Cricket Protein

**DOI:** 10.3390/foods11030337

**Published:** 2022-01-25

**Authors:** Cristhiam E. Gurdian, Damir D. Torrico, Bin Li, Witoon Prinyawiwatkul

**Affiliations:** 1 School of Nutrition and Food Sciences, Louisiana State University, Agricultural Center, Baton Rouge, LA 70803, USA; cgurdi3@lsu.edu; 2 Department of Wine, Food and Molecular Biosciences, Faculty of Agriculture and Life Sciences, Lincoln University, Lincoln 7647, New Zealand; damir.torrico@lincoln.ac.nz; 3 Department of Experimental Statistics, Louisiana State University, Agricultural Center, Baton Rouge, LA 70803, USA; bli@lsu.edu

**Keywords:** emotion-based liking, alternative protein, consumer behavior, purchase intent, perceptions, holistic sensory analysis

## Abstract

Sustainable and nutritious alternatives are needed to feed the ever-increasing world population. The successful incorporation of edible-cricket protein (ECP) into foods needs deeper consumer insights. Treatments (plain, Italian, and Cajun pita chips containing 6.9% *w/w* ECP) were evaluated by subjects for overall liking (OL), emotions, and purchase intent (PI) in three different moments: (1) before tasting, (2) after tasting/before ECP statement, and (3) after tasting/after ECP statement. Attributes’ liking scores were evaluated only after tasting/before ECP statement. Liking scores (mixed-effects ANOVA), emotions, and PI across moments within treatments/across treatments within moments were evaluated. Emotion-based penalty-lift analyses for OL within moments were assessed using two-sample *t*-tests (*p* < 0.05). Random forest model analyzed after-tasting informed PI and variables’ importance. Although formulations’ OL and PI were similar across moments, plain and Italian chips had higher after-tasting (before and after ECP statement) OL than the Cajun chips. Moments indirectly affected OL via emotions elicitation. Valence and activation/arousal emotions discriminated across moments for the plain treatment whereas valence and mostly activation/arousal terms discriminated across moments for the Italian and Cajun treatments, respectively. For either formulation or moment, “interested” and “adventurous” positively affected OL. Before and after-tasting attribute liking, “satisfied,” and “enthusiastic” emotions were critical in predicting after-tasting informed PI.

## 1. Introduction

The expected increase in the global population accompanied by increasing rates of the depletion of natural resources, such as water and land, urgently calls for innovative changes in the actual food supply [1]. In addition, consumers’ demand for more sustainable food products that still meet their expectations regarding nutrient and hedonic profiles continues to rise [2]. Edible cricket protein (ECP) is considered among the most acceptable insect-derived ingredients in the Western world [3,4]. Besides imparting the desired functionality as a protein ingredient, ECP imparts a higher quality nutritional profile [5] to foods without significantly changing the sensory acceptability [6,7]. However, achieving this goal is highly dependable on how ECP products are perceived in terms of their sensory characteristics. Moreover, ECP products’ acceptability can be affected by the disclosed information, demographic factors (e.g., gender, age, and race [1,8,9]), and other consumer-niche variables [10]. The level at which ECP can be incorporated into foods varies greatly depending on the food application [1,6,7,11,12,13]. Changes in the flavor profile is the most cited limiting constraint/factor for ECP [5], followed by modifications in color and/or texture [1,6,7,11,13]. In a recent study, Ardoin et al. [14] found that 15% of ECP in crackers produced a 20% rejection rate in consumers and thus recommended 7.9% as the upper level for its addition into foods.

Several studies have suggested strategies (e.g., familiarity tasting approach, influencers adoption broadcasting, invisible ingredients, educational sessions, etc.) to improve the adoption of insect-derived ingredients [8,9,15]. In addition, consumers’ acceptability can be influenced by extrinsic factors [16,17], including packaging [18], benefits statements [19], serving inputs [20], and vestibular sensations [21]. Moreover, ECP incorporations faces psychological constraints [1,8], which are accentuated in Western cultures [22]. Food neophobia [4] and disgust sensitivity have been identified as the major limitations of foods containing insect-derived ingredients in Western societies [23,24,25]. Despite this, food neophobia can be overcome through familiarization of the consumers with entomophagy mainly through repeated exposure to successful tasting experiences. However, suppressing the disgust sensation towards edible insect ingredients represents a more challenging task for new product formulators.

In Western cultures, the ultimate success of foods containing edible insect ingredients will indeed be driven by consumer behaviors. Hence, it has been proposed that, instead of attempting to convince unwilling consumers to experience edible insects, the efforts and strategies in products formulated with edible insects should be aimed at improving the eating experience for potential early adopters [9] and identifying the market niche for these types of products [1,8]. Potential adopters of products formulated with ECP or other insect-derived ingredients seem to have a consistent pattern of sensations and emotions when experiencing products containing edible insects. Hence, these so-called sensation-seeker perceptions, expectations, liking, and behaviors should then be further studied to obtain meaningful insights for the incorporation of products containing edible insects in Western cultures.

Previous research involving consumers’ attitudes towards edible insects suggests that food-evoked emotions in addition to hedonic ratings, product liking, and other descriptive and experimental components shall be considered to achieve holistic models that accurately predict and interpret consumer behaviors [8,26,27]. Therefore, the aim of this study was to explore the effect of tasting and informing an ECP statement on the hedonic perceptions, evoked sentiments, and PI of three pita chips formulations containing 6.9% *w/w* ECP (plain, Italian, and Cajun) in three moments: (1) before tasting, (2) after tasting/before ECP statement, and (3) after tasting/after ECP statement). Likewise, the formulations’ effect on hedonic perceptions and emotional patterns as they relate to consumer behaviors (PI) was investigated. The associations among variables (consumer, product, and experimental) were explored to provide meaningful insights for the development of novel foods containing ECP.

## 2. Materials and Methods

### 2.1. Pita Chips Preparation

Whole wheat Gold Medal flour (General Mills Sales, Inc., Minneapolis, MN, USA), Morton lite salt (Morton Salt, Inc., Chicago, IL, USA), Great Value double-acting baking powder, purified drinking water, and Sam’s Choice Italian style herb (basil, marjoram, oregano, rosemary, sage, and thyme) grinder seasoning (Great Value, Wal-Mart Stores, Inc., Bentonville, AR, USA), Slap Ya Mama low sodium Cajun seasoning (Walker & Sons, Inc., Ville Platte, LA, USA), and McCormick sundried tomato basil pasta sauce and seasoning mix (McCormick & Co., Inc., Hunt Valley, MD, USA) were purchased at Walmart Supercenter (Baton Rouge, LA, USA). Organic triple filtered coconut oil (Trader Joe’s, Monrovia, CA, USA) was purchased at Trader Joe’s grocery store and Thailand unique microwave-dried edible cricket protein (ECP, JR Unique Foods Ltd., Udon Thani, Thailand) made of 100% farmed and powdered house crickets (*Acheta domesticus*) was purchased online from www.amazon.com (accessed on 28 August 2020). Treatments’ seasonings (plain, Italian, and Cajun) were selected in consensus from a focus group session with *n* = 15 pita chips consumers and a skilled moderator considering available information from recipes, magazines, and personal cooking experience followed by a preliminary trial evaluating overall acceptability using a 9-point hedonic scale and open-ended questions to relate back to product development in bench (e.g., what would you change in this product?) with the same subjects. Batches of treatments (plain, Italian, and Cajun pita chip formulations) were prepared by hand mixing the ingredients (Table 1), followed by kneading to produce dough, refrigerated (4 °C) resting for 30 min, rolling, and shaping (triangular shape). Then, raw chips were placed in 45.7 cm × 66 cm aluminum trays and baked in a pre-heated OV310G mini rotating rack oven (Baxter Mfg, a Division of ITW FEG, LLC, Orting, WA, USA) at 325 °F for 20 min. Baked treatments (plain, Italian, and Cajun pita chips containing 6.9% *w/w* ECP) were stored in separate containers at room temperature overnight (12 h approximately) until the consumer test was performed. The ECP concentration (6.9% *w/w*) added to the treatments was selected based on the recommendations from previous research [1,5,7,11,12,13] and a preliminary trial with *n* = 20 subjects, who were consumers of pita chips and indicated overall acceptability scores of at least 5.5 on a 9-point hedonic scale.

**Table 1:** Formulation of pita chip treatments ^†^.

Ingredients	Plain		Italian		Cajun	
	Amount (g)	Amount (%)	Amount (g)	Amount (%)	Amount (g)	Amount (%)
Whole wheat flour	36.78	45.98%	35.60	44.50%	34.68	43.35%
Purified drinking water	33.04	41.30%	33.04	41.30%	33.04	41.30%
ECP	5.52	6.90%	5.52	6.90%	5.52	6.90%
Coconut oil	3.67	4.59%	3.67	4.59%	3.67	4.59%
Lite salt	0.70	0.88%	0.70	0.88%	0.70	0.88%
Baking powder	0.29	0.36%	0.29	0.36%	0.29	0.36%
Cajun seasoning	-		-		2.10	2.63%
Sundried tomato basil seasoning	-		0.92	1.15%	-	
Italian-style herb seasoning	-		0.26	0.33%	-	

^†^ Treatments are described in Figure 1. ECP = Edible cricket protein.

**Figure 1:**
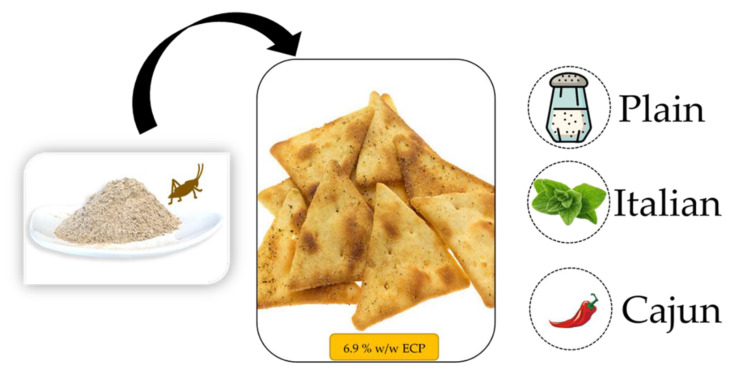
Graphical description of treatments (plain, Italian, and Cajun pita chip formulations containing 6.9 % *w/w* ECP). ECP = Edible cricket protein.

### 2.2. Sensory Evaluation

#### 2.2.1. Panelists

The research protocol for this study was approved by the Louisiana State University (LSU) Agricultural Center Institutional Review Board (IRB # HE 18-9). Untrained participants (*n* = 84) 18 years of age and older (Table 2) were recruited from the LSU campus, Baton Rouge, LA, USA, on 10 November 2020 and subsequently screened. The number of participants for this study was low because the study took place during the early stage of the COVID-19 pandemic and social distance in addition to other restrictions and precautions prevented the study from recruiting more participants. The screening criteria consisted of: (1) no allergies or adverse reactions toward any ingredient of the samples or unsalted crackers, (2) willingness to taste products containing edible cricket protein (ECP), (3) no indication of conditions that may impair their performance as panelists, and (4) being regular consumers (at least once per month) of pita chips.

#### 2.2.2. Consumer Study

On the day of the study, pita chip treatments (plain, Italian, and Cajun formulations) were placed (6 chips per treatment) inside Great Value (5.1 × 4.7 in) clear-plastic square snack zipper bags (Wal-Mart Stores, Inc., Bentonville, AR, USA) labeled with three-digit random codes. All participants received the three treatments in different moments (before tasting, after tasting before ECP statement, and after tasting after ECP statement) in one session. The consumer study was conducted in sanitized (before each subject’s session) partitioned booths. Each booth was separated from another by a 6 ft distance to comply with the COVID-19 precautions established in place at LSU. The sensory booths were equipped with white lights and the room was kept at 25 °C.

#### 2.2.3. Questionnaire

Participants’ responses were collected with the online Qualtrics software version 11.2020 (Qualtrics, Provo, UT, USA) accessed on 1–10 November 2020. Before evaluating the treatments (Figure 1), subjects agreed with and signed a consent form included in the approved research protocol. Then, demographic information (gender, age, and race) and whether a previous edible insect consumption had occurred was recorded from the participants. The three pita chip treatments were presented together, but panelists were instructed to evaluate them in a monadic sequential order as indicated on the screen. Participants were asked to clean their palate with unsalted crackers and water before tasting the first sample and in between samples. The treatments’ evaluation followed a balanced randomized complete block design

For a given treatment, first, panelists were instructed to evaluate the sample before tasting (moment 1) for: (1) aroma (smell) and overall liking (OL) with a 9-point hedonic scale (1 = dislike extremely and 9 = like extremely), (2) purchase intent (PI) with a binomial (yes or no) scale, and (3) evoked emotions with a check-all-that-apply (CATA) task using twenty-five emotion terms obtained from the Essense25 list [28]. Then, panelists were instructed to taste the sample (moment 2) and evaluate crunchiness, overall flavor, OL (9-point hedonic scale), PI (binomial scale), and evoked emotions using CATA (Essense25). Finally, the questionnaire displayed the following statement regarding the ECP used in the formulations: “Edible insects are safe to eat and are considered a sustainable source of high-quality protein and other nutrients. Edible insect production has less negative environmental impact than traditional livestock production. An estimated 2 billion people worldwide consume edible insects”, and panelists were instructed to evaluate again the sample (moment 3) for OL (9-point hedonic scale), PI (binomial scale), and evoked emotions using CATA (Essense25).

### 2.3. Data Analysis

The evaluation of the pita chip treatments (Figure 1) followed a balanced randomized complete block design (panelists as blocks). The R software version 4.0.3 (RStudio, Inc., Boston, MA, USA) [29] and the XLSTAT version 2019.3.1 (Addinsoft, New York, NY, USA) statistical software version 2020 [30] with α = 0.05 significance level were used for the data analysis. The effect of demographics, formulation (plain, Italian, and Cajun), moment (before tasting, after tasting before ECP statement, and after tasting after ECP statement), two-way interactions (gender (females vs. males) * previous edible insect consumption (yes vs. no) and previous edible insect consumption * formulation), and interactions up to three-way (gender * formulation * moment) on sensory likings (excluding moment) and on overall liking (OL) were investigated with multi-way analysis of variance (ANOVA) in a mixed-effects model having panelists as a random effect and Tukey’s HSD post-hoc test. Two-sided Cochran’s Q test followed by asymptotic McNemar test for post hoc multiple pairwise comparisons [31] with *p*-value adjusted by false discovery rate [32] was used to compare the frequencies of PI = “Yes” across moments (before tasting, after tasting before ECP statement, and after tasting after ECP statement) within treatments (plain, Italian, and Cajun) and across treatments within moments. Check-all-that-apply (CATA) binary data from evoked emotions were analyzed according to Meyners et al. [33] and Ares et al. [34] across moments within treatments and across treatments within moments. First, Cochran’s Q tests determined the overall and individual effect of moments within treatments and treatments within moments in emotions list distribution and each emotion term frequency distribution, respectively. Subsequently, all pairwise comparisons were performed for moments as well as treatment groups following the Marascuilo and McSweeney method [35]. For each treatment, emotions, moments, and PI were then visualized with a correspondence analysis based on chi-square distances. For each moment (pooling treatments together), the relationship (drivers/inhibitors) between evoked emotions and product liking was studied through penalty-lift analysis of OL. Overall-liking mean impact was calculated as the mean OL difference from present vs. absent categories for each emotion with a 20% population threshold [36]. This difference was then standardized, and its significance (*p* < 0.05) was tested with a two-sample *t*-test. Finally, a random forest (RF) algorithm ensembled and combined *n* = 1000 decision trees to predict after-tasting (after ECP statement) PI [37] using mtry = 6 features out of 36 in the random selection at each splitting node of the *n* = 1000 decision trees. Formulation, population variables, OL from each moment, and after-tasting sensory likings and emotions were input to the RF algorithm using full data as the main interest on model interpretation. The misclassification rate for RF was calculated using the out-of-bag observations, which provide a cross-validation-like estimation of the prediction accuracy, and the classifier’s performance was plotted on the receiver operating characteristic (ROC) curve. The variables’ contribution for the prediction of the response variable in an RF model can be determined by two approaches: (1) the mean decrease in prediction accuracy after permuting each predictor on the out-of-bag samples, and (2) the mean decrease in the Gini index, which measures node impurity for classification trees, from splitting on the variable and averaging over the trees in the RF ensemble.

## 3. Results and Discussion

### 3.1. Overall Significance of Main Effects on Product Liking

**Figure 2:**
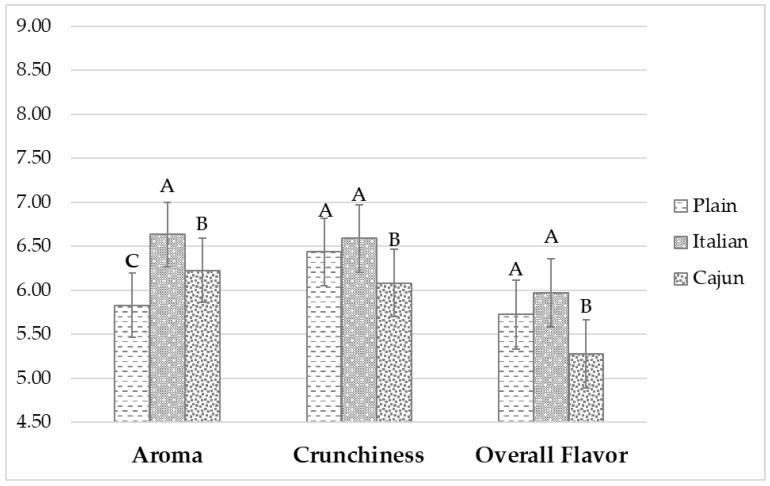
Treatments’ sensory acceptability bar chart for the before-tasting (aroma) and after-tasting (before edible cricket protein (ECP) statement) moments (crunchiness and overall flavor). Data are liking ratings least square means and standard errors from *n* = 84 consumers. Treatments are described in Figure 1. Different uppercase letters indicate significantly (*p* < 0.05) different liking scores (Tukey’s means separation) across treatments.

**Figure 3:**
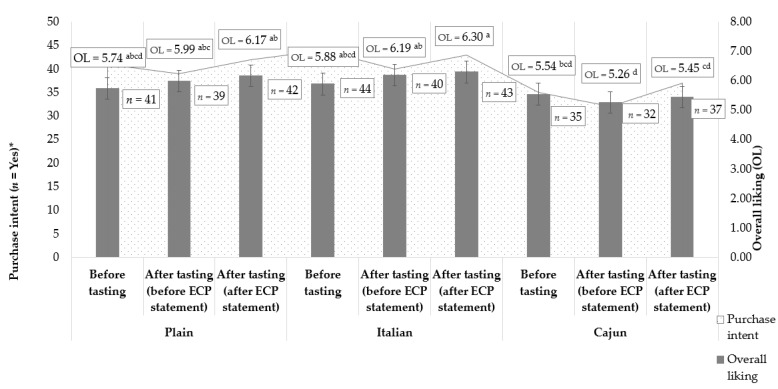
Treatments overall liking (OL; grey bars) and purchase intent (PI; trend line) chart comparing formulations and moments (before tasting, after tasting, and after edible cricket protein (ECP) statement). Data are OL least square means and standard errors/frequencies of PI = Yes from *n* = 84 consumers. Treatments are described in Figure 1. Different lowercase letters indicate significantly (*p* < 0.05) different OL scores (Tukey’s means separation) across treatments and moments. * No significant (*p* > 0.05) difference in PI frequencies across treatments within a given moment or across moments within a given treatment (Cochran’s Q test followed by asymptotic McNemar test for post hoc multiple pairwise comparisons and *p*-value adjustment by false discovery rate).

The significance of the main effects and their two and three-way interactions of interest on the sensory acceptability of treatments (Figure 1) are presented in the analysis of variance (ANOVA; Table 3). Formulation and its two-way interaction with gender were significant (*p* < 0.05) effects for all the sensory attributes. Disregarding all other effects, the liking scores for aroma (plain = 5.83, Italian = 6.63, Cajun = 6.22), crunchiness (plain = 6.43, Italian = 6.59, Cajun = 6.08), overall flavor (plain = 5.72, Italian = 5.97, Cajun = 5.28), and overall liking (OL, plain = 5.97, Italian = 6.13, Cajun = 5.42) were different depending on the formulation (Figures 2 and 3). Gender levels (female vs. male) influenced the way subjects rated their liking depending on the formulation levels (plain, Italian, Cajun) for aroma, crunchiness, overall flavor, and OL (Table 3). Females’ liking scores for aroma were higher than those of males but the liking scores for crunchiness, overall flavor, and OL were higher for males than for females (data not shown). These results agree with the findings from other studies involving edible cricket protein (ECP) for which females exhibited lower taste thresholds and blind acceptability upon tasting for products formulated with ECP than males [1,38]. However, under the conditions of this study, females may have exhibited a higher rejection threshold for the aroma or smells (before tasting) imparted by ECP than males. Previous edible insect consumption significantly (*p* < 0.05) interacted with the formulation effect causing the treatments’ aroma (before tasting) acceptability to be mediated by previous edible insect experience (Table 3). Overall, subjects who had experienced edible insects had a lower aroma liking score for all the treatments except for the Cajun formulation (plain with edible insects experience = 5.69 vs. plain without edible insects experience = 5.96; Italian with edible insects experience = 6.54 vs. Italian without edible insects experience = 6.73; Cajun with edible insects experience = 6.58 vs. Cajun without edible insects experience = 5.87; data not shown in Tables or Figures). Findings from the aroma liking for the Cajun formulation agree with other studies reporting higher sensory likings for products formulated with edible insects when subjects had experienced them before [8,25,39].

### 3.2. Effects of Formulation on Sensory Acceptability of Treatments

Figure 2 shows the sensory acceptability of the treatments (Figure 1) for aroma (before tasting) and after-tasting (before ECP statement) crunchiness and overall flavor. The Italian formulation had the highest (*p* < 0.05) aroma liking followed by the plain formulation, which obtain a significantly (*p* < 0.05) higher aroma liking than the Cajun formulation. However, for crunchiness and overall flavor likings, the Italian and plain treatments presented similar ratings, which were significantly (*p* < 0.05) higher than that for the Cajun treatment. These results suggest that the seasonings used for snacks with incorporations of ECP have an effect on consumers’ acceptability. More extravagant or accentuated flavors like the spices contained in the Cajun treatment may become more acceptable in a later stage of the life cycle of products formulated with ECP but are not yet attractive in an introductory phase [40]. In addition, the observed results show the positive effect of introducing new ingredients, such as ECP, through familiar products [1,11,41] and flavors (flavor notes imparted by the Cajun treatment may not be familiar to pita chips consumers).

### 3.3. Effects of Moment on Overall Product Liking and Purchase Intent

Figure 3 depicts the overall liking (OL) and purchase intent (PI) of the treatments in the before-tasting and after-tasting (before and after the ECP statement) moments. Although moment did not significantly (*p* > 0.05) affect the overall liking (OL) nor the purchase intent (PI) for any of the treatments, the OL within the after tasting moments (before and after the ECP statement) differed across the treatments. Plain and Italian formulations presented higher (*p* > 0.05) OL ratings than the Cajun formulation in both after-tasting moments. These results agree with the observed higher liking ratings for these two formulations for after-tasting (before ECP statement) crunchiness and overall flavor, which suggests that the liking of these two attributes [11] influenced ultimate product liking [20] more than other sensory attributes or product benefit statements. No PI differences across formulations were observed within each moment either. This suggests that other orthogonal variables to the sensory dimension, such as emotions and sensations, may also affect PI and consumer behavior [26,42,43,44]. The non-significant effect of the ECP statement in the OL and PI of treatments observed in this study agrees with the findings from other studies investigating the psychological traits behind the reluctance to consume edible insects [45,46,47]. Possibly, for products formulated with edible insects, environmental [24,48] or health-related statements about entomophagy are insufficient to significantly improve their sensory liking profile [46,49]. However, the OL, which was not disconfirmed upon tasting, and PI achieved by the plain and Italian treatments indicate an overall positive marketing potential [50] as they represent a new concept for the Western consumers, and their formulations could be further optimized to achieve higher sensory liking [1].

### 3.4. Discriminative Effects of Moments and Formulation on Treatments’ Emotional Profile

The emotional profile of treatments was segmented and evaluated across moments within formulations and across formulations within moments (Table 4, Table 5 and Table 6).

#### 3.4.1. Emotional Profiles across Moments

**Figure 4:**
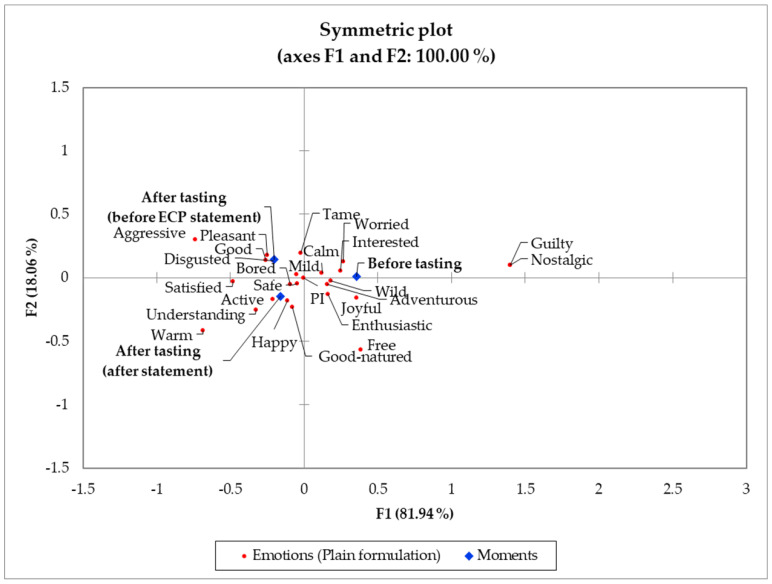
Correspondence analysis (chi-squared distance) symmetric plot visualizing the plain treatment moments (before tasting, after tasting, and after edible cricket protein (ECP) statement) and emotions from *n* = 84 consumers. Treatments are described in Figure 1.

Figure 4 depicts the symmetric plot of elicited emotions and moments (before tasting, after tasting before ECP statement, and after tasting after ECP statement) for the plain treatment. Tasting had a more pronounced effect than the ECP statement in discriminating the evoked emotions for the plain formulation. For the before-tasting moment, the “worried” (extreme activation/arousal) and “interested” (moderate activation/arousal) emotions were associated with the plain treatment. Possibly subjects’ worry arose from a safety (health-related context) concern because of the limited information regarding edible insects’ regulations and processes that guarantee their safe use as ingredients for human foods [8,51,52,53,54]. Yet, their curiosity and interest may have been triggered by the fact that the treatments contained ECP and the tasting experience they were about to have [24,55,56].

Both after-tasting moments (before and after the ECP statement) were associated with positive-valence emotions such as “good” and “pleasant” (before the ECP statement) and “happy” and “good-natured” (after the ECP statement). However, the “disgusted” term was also highly associated with the after-tasting before ECP statement moment while the “active” high activation/arousal term was more associated with the after-tasting after ECP statement moment for the plain treatment (Figure 4).

Ultimate product acceptability is positively associated with positive-valence emotions [26,27,49] and negatively associated with “disgust” feeling, which can vary across genders [57]. For novel products formulated with edible insects, sensation-seeking emotions, such as “active” should be elicited as they are important predictors of product acceptability [24,58], and previous research has emphasized that the context for launching products formulated with ECP should encompass novelty, adventure, and wild features [8,43].

On the other hand, the “safe” (positive valence and low activation/arousal) and “bored” (low activation/arousal) were closely positioned to both after tasting moments (before and after the ECP statement). The pattern of emotions occurrence belonging to both positive and negative valences in the pleasantness dimension and both high and low activation/arousal dimension suggests that consumers belonged to different segments [59]. However, the pattern of emotions should be interpreted with caution as not all terms occurrences may be significant.

Table 4 shows the frequency distribution for the plain treatment emotions. Both valence and activation/arousal emotions equally contributed to the differentiation across moments, having only six (“adventurous,” “aggressive,” “good,” “interested,” “satisfied,” and “understanding”) significant emotions. The “adventurous” term frequency decreased upon tasting, but, after showing the ECP statement, it increased to a similar level than before tasting [60]. Although the “aggressive” emotion was more elicited (*p* < 0.05) after tasting (before the ECP statement) than before tasting, the compared frequencies are sparse and may not indicate any practical differences. On the other hand, the ECP statement was not effective in achieving the initial interest level for the plain treatment, which decreased upon tasting. This could reflect a partial (if not total) disagreement with the ECP statement [61]. Regarding emotions lying in the pleasantness dimension, “satisfied” and “understanding” were more elicited (*p* < 0.05) after tasting before the ECP statement and after tasting after the ECP statement, respectively, than before tasting [62]. However, for the “good” emotion term, tasting significantly increased its frequency of occurrence, but disclosing the ECP statement after tasting caused a slight reduction in its frequency.

**Figure 5:**
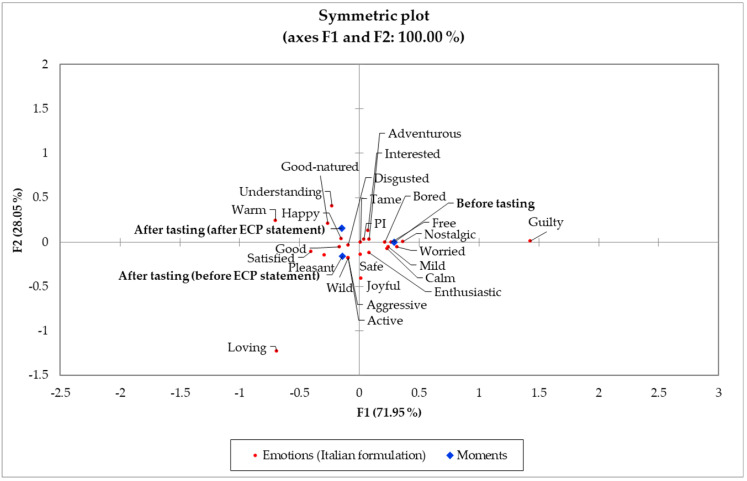
Correspondence analysis (chi-squared distance) symmetric plot visualizing the Italian treatment moments (before tasting, after tasting, and after edible cricket protein (ECP) statement) and emotions from *n* = 84 consumers. Treatments are described in Figure 1.

Figure 5 depicts the symmetric plot of elicited emotions and moments (before tasting, after tasting before ECP statement, and after tasting after ECP statement) for the Italian treatment. Again, the effect of tasting was larger than that of the ECP statement. For the before tasting moment, the “worried” (extreme activation/arousal), “mild,” “calm,” and “bored” (low to moderate activation/arousal), “free,” and “nostalgic” [63] (positive) emotions were associated with the Italian treatment. While the worry and concern about the safety of ECP are very likely to negatively affect product liking and consumption [64], the Italian formulation may benefit from the low to moderate activation/arousal elicited emotions as they have been found to increase appetite and food intake [65]. The other positive emotions have an overall positive effect on product liking and consumption that has been well documented, although some studies report a weak contribution of the “nostalgic” emotion [27,42].

For both after-tasting moments (before and after the ECP statement), an association with positive-valence emotions such as “good” and “happy” but also with the negative “disgusted” term was observed for the Italian treatment (Figure 5), suggesting that elicited emotions for this treatment varied across subjects, possibly because there were consumers from different segments [59]. On the other hand, the Italian formulation was strongly associated with the sensation-seeking emotions “wild,” “aggressive,” and “active” with regard to both dimensions (F1 and F2). This reflects a positive effect of tasting for the Italian treatment as these emotions lie on the high activation/arousal dimension, which, together with liking and emotions that belong to the pleasantness dimension, have strong predictive power for consumer behavior and positively affect product consumption willingness [8,9,24,58].

Table 5 contains the frequency of the emotional responses for the Italian treatment. Only positive-valence emotions (“good-natured,” “pleasant,” “satisfied,” and “understanding”) were significant in discriminating across moments. Overall, tasting and the ECP statement had a positive effect on the Italian treatment as positive-valence terms were more evoked (*p* < 0.05) upon tasting than before tasting (“pleasant” and “satisfied”) [62] or upon disclosing the ECP statement (“good-natured” and “understanding”) [8].

**Figure 6:**
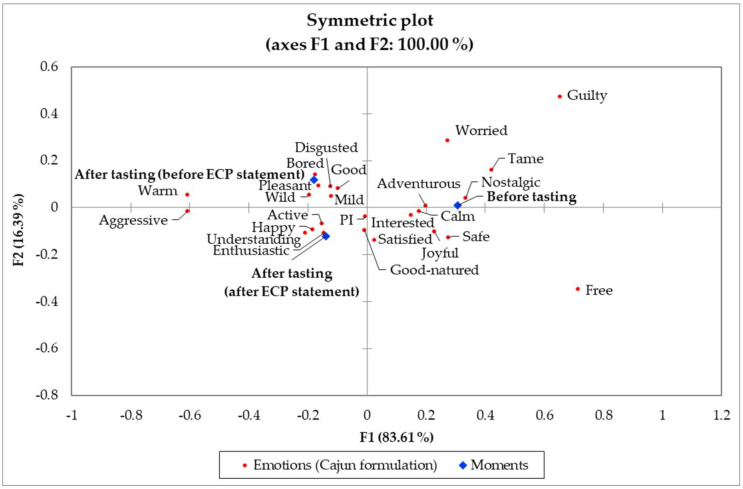
Correspondence analysis (chi-squared distance) symmetric plot visualizing the Cajun treatment moments (before tasting, after tasting, and after edible cricket protein (ECP) statement) and emotions from *n* = 84 consumers. Treatments are described in Figure 1.

Figure 6 contains the symmetric plot of evoked emotions and moments (before tasting, after tasting before ECP statement, and after tasting after ECP statement) for the Cajun treatment. Like the other treatments, the tasting effect was larger than the ECP statement effect in the emotional profile across moments. For the before-tasting moment, the Cajun treatment was mainly associated with the “nostalgic” term, which has been categorized as a driver of sensory pleasure [63] but also as a neutral term [42], and to a lesser extent with the “adventurous” term, indicative of an active and energetic state [8,64].

Regarding Cajun treatment’s after-tasting moments, the before ECP statement was highly associated with both high and low to moderate activation/arousal emotions (“wild” and “bored”, respectively) [66] and with the positive-valence “pleasant” term and, to a lower extent, with the negative “disgusted” term (Figure 6). As previously mentioned, this could reflect the need for further consumer segmentation [59], but it could also indicate mixed feelings (consumer ambivalence) [67] toward the entomophagy concept, which is still an unfamiliar practice for Western cultures [68]. Except for “disgusted”, the close association with the other terms may be beneficial for the Cajun treatment as evoking sensations and feelings that belong to the activation/arousal and pleasantness dimensions is a positive effect for novel foods and ingredients such as ECP [8], and “bored” does not necessarily negatively impact food consumption [65]. On the other hand, when the ECP statement was disclosed, the “active” and “enthusiastic” emotions belonging to the high activation/arousal dimension characteristic of the sensation seekers [69] were highly associated with the Cajun formulation as was the positive-valence “happy” emotion. This suggests that the ECP statement had a positive effect maintaining the sensation-seeking and pleasant emotions while distancing from the “disgusted” negative emotion. Disgust feeling is among the top constraints to entomophagy in Western cultures [24,70]; therefore, it is a key sensation yet to be further investigated to find ways to minimize or prevent it from being elicited in foods formulated with edible insect ingredients [71,72]. However, the significance of each term frequency will determine their ultimate effect in discriminating among the treatments, moments, and their impact on product liking.

Table 6 presents the frequency of emotions for the Cajun treatment exploring the effects of tasting and ECP statement. As for the other treatments, emotions distribution significantly (*p* < 0.05) varied across moments, but, for this formulation, mostly activation/arousal terms discriminated across moments. The frequency of the “adventurous” emotion decreased (*p* < 0.05) upon tasting compared to before tasting regardless of the ECP statement, but, for the “aggressive” term, it was increased upon tasting regardless of the ECP statement. The “adventurous” emotion is characteristic of the sensation seekers [69] market niche and belongs to the activation/arousal dimension [73,74], which has been suggested as the appropriate context for the introduction of ECP [8]. Yet, the “aggressive” term has been classified as an arousal [64], neutral [42], and as a negative [73] term. Hence, this effect needs to be further investigated for its relationship with product liking and the willingness to consume or to purchase the product. A positive effect of the ECP statement was observed for the “interested” and “worried” emotions. The first became less evoked (*p* < 0.05) upon tasting than before tasting but increased to a similar level than before tasting after disclosing the ECP statement. The second became less frequent (*p* < 0.05) when the ECP statement was disclosed when compared to the before-tasting moment. Finally, tasting increased the “warm” emotion frequency, which remained constant after disclosing the ECP statement. 

However, this may represent the “warm” taste sensation imparted from the spiciness of the Cajun formulation rather than a “warm” feeling elicited by this treatment.

#### 3.4.2. Emotional Profiles across Formulations

For the before-tasting moment, the overall hypothesis testing whether the distribution of emotions differed across formulations could not be rejected at the *p* = 0.05 confidence level. However, the Italian formulation presented a significantly lower frequency of the “adventurous” emotion than the other two formulations. “Adventurous” term is an important emotional attribute ideally evoked before and after tasting because it belongs to the sensation-seeking emotions, which have strong predictive power and are drivers of liking for novelty products formulated with ECP [24,43,64].

For both after-tasting moments (before and after the ECP statement), the distribution of emotions varied (*p* < 0.05) across formulations. For the after-tasting (before ECP statement) moment, the “enthusiastic” term belonging to the pleasantness and activation/arousal dimensions was significantly (*p* < 0.05) lower for the plain treatment when compared to the Cajun treatment while the “satisfied” positive-valence emotion was significantly lower for the Cajun treatment when compared to the Italian treatment. Possibly the variety of flavors and hot/spicy sensations from the Cajun formulation contrasted with the plain formulation flavor, making the subjects feel more “enthusiastic” about the Cajun flavor notes [74]. The observed decreased frequency in the “satisfied” term for the Cajun treatment suggests that this emotion is an important driver of product liking, possibly leading to the observed lower OL for the Cajun formulation in the after-tasting (before ECP) moment [8]. In addition, the “warm” emotion was significantly (*p* < 0.05) more elicited for the Cajun treatment when compared to the plain and Italian formulations in both after-tasting moments (before and after the ECP statement) but, as previously mentioned, this could reflect the “warm” sensations imparted from the spicy flavor notes in the Cajun formulation. On the other hand, for the after-tasting (after ECP statement) moment, the plain and Italian formulations had lower (*p* < 0.05) frequencies of the “aggressive”, “enthusiastic” (active and pleasant) [75], and “warm” (pleasant) [42] terms than the Cajun formulation. However, as previously mentioned, the “aggressive” term does not have a definite valence, its impact on product liking varies greatly across studies, and the “warm” term may just reflect the spiciness perception for the Cajun formulation. Hence, the observed differences in the “enthusiastic” emotion frequency across formulations may be attributed to a more varied flavor profile imparted by the Cajun treatment when compared to the plain and Italian treatments.

### 3.5. Elicited Emotions and Product Liking

The effect of emotions elicited at each moment (before tasting, after tasting before ECP statement, and after tasting after ECP statement) on overall product liking (disregarding the formulation effect as it was shown to be minimal within each moment emotional profile) was investigated by calculating overall liking (OL) mean impacts.

**Figure 7:**
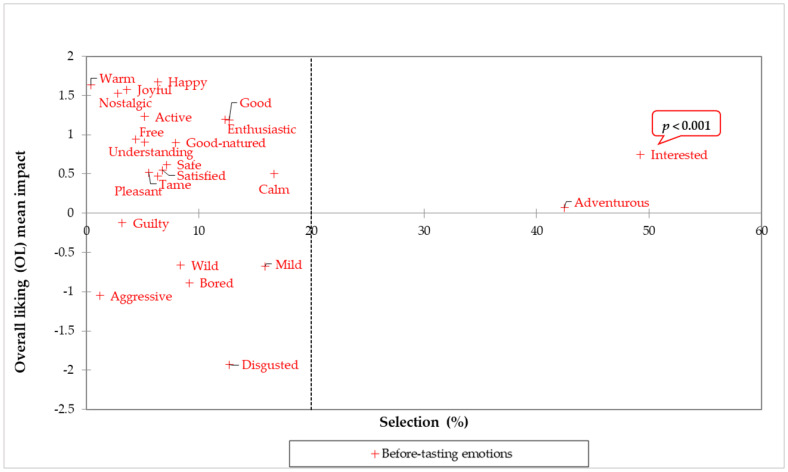
Treatments’ before-tasting overall liking (OL) mean impact (mean OL difference from present vs. absent categories for each emotion with a 20% population threshold size) vs. significant (*p* < 0.05, 2-sample *t*-test) emotions in the before-tasting moment (%) from *n* = 84 consumers. Before-tasting emotions and OL from treatments (Figure 1) were pooled together for the analysis.

Evoked emotions in the before-tasting moment and their respective OL mean impact (increase or decrease) from all the three treatments (Figure 1) are presented in Figure 7.

**Figure 8:**
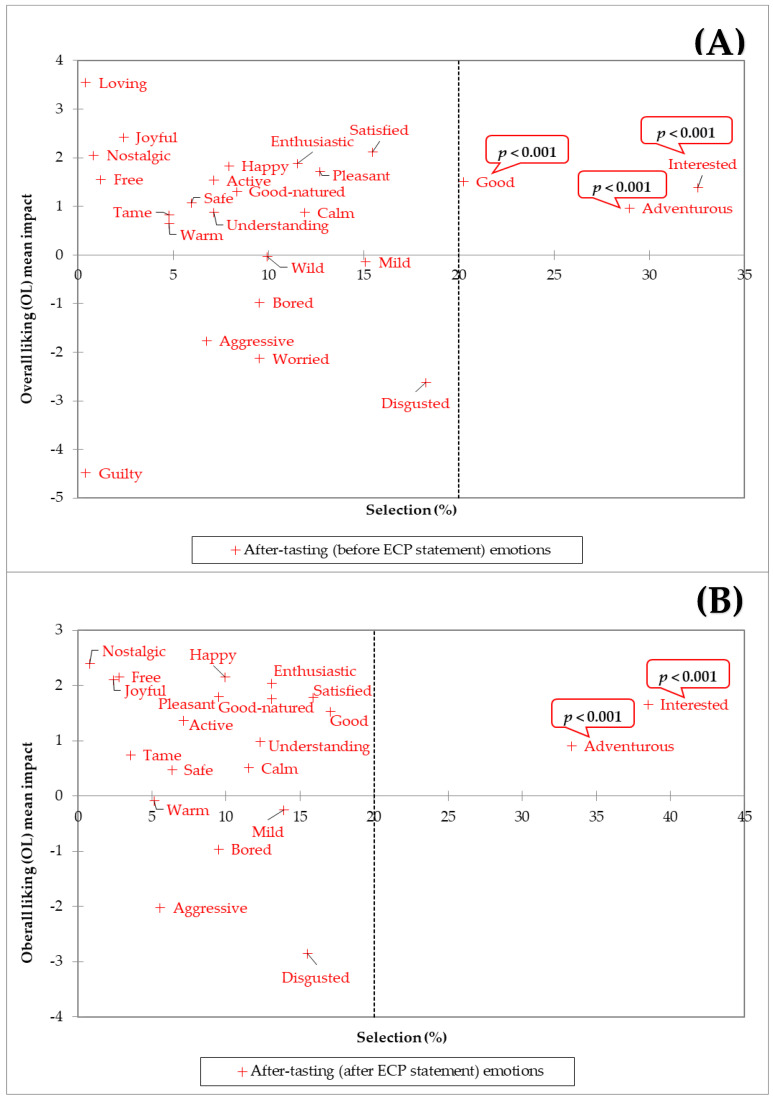
Treatments’ after-tasting overall liking (OL) mean impact (mean OL difference from present vs. absent categories for each emotion with a 20% population threshold size) vs. significant (*p* < 0.05, 2-sample *t*-test) emotions in the after-tasting moment (%) from *n* = 84 consumers (**A**) before edible cricket protein (ECP) statement and (**B**) after ECP statement. After-tasting emotions and OL from treatments (Figure 1) were pooled together for the analysis.

For the before-tasting moment, only the “interested” emotion caused a significant (*p* < 0.001) increase in the treatments’ OL, whereas “good,” “adventurous, and “interested” were all significant (*p* < 0.001) drivers for the after-tasting (before ECP statement) OL of the treatments (Figure 8A). These results agree with other studies highlighting the importance of evoking sensation-seeking emotions and positive-valence emotions for expected and actual product liking in foods containing edible insects [8]. The increased experience of positive-valence emotions upon tasting, such as “good”, has been widely documented in research with foods [76], but the continued frequency across moments of significant sensation-seeking emotions seems to be specific for foods, such as spicy foods [77], and those lying in the context of novelty and adventure, like edible insects. Unexpectedly, when the ECP statement was shown, “good” was no longer a significant driver of product liking because its frequency (pooled across treatments) was reduced, falling below the user-defined 20% selection threshold (Figure 8B). Possibly, although the ECP statement contained environmental and health benefits information associated with entomophagy, subjects may have experience disagreement with some (if not all) the information communicated [61]. On the other hand, it is also possible that the statement triggered a reminder of mental associations that decreased the “good” emotion of the participants from this study [78,79,80].

### 3.6. Purchase Intent (PI) Prediction and Variables’ Importance

**Figure 9:**
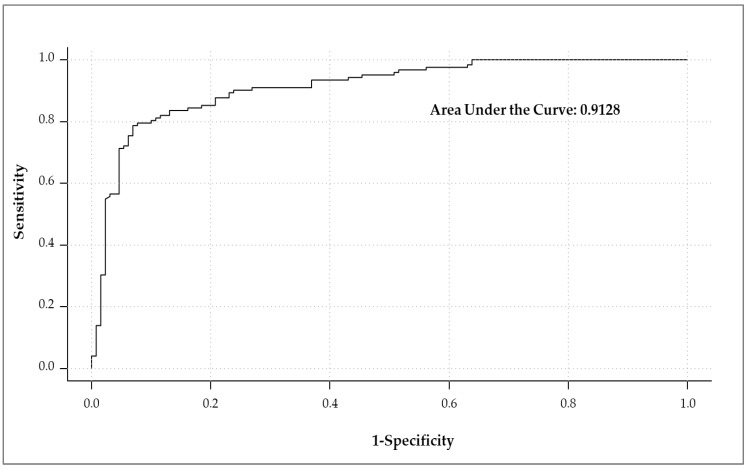
Receiver operating characteristic (ROC) curve illustrating the area under the curve (AUC) for the random forest classifier.

**Figure 10:**
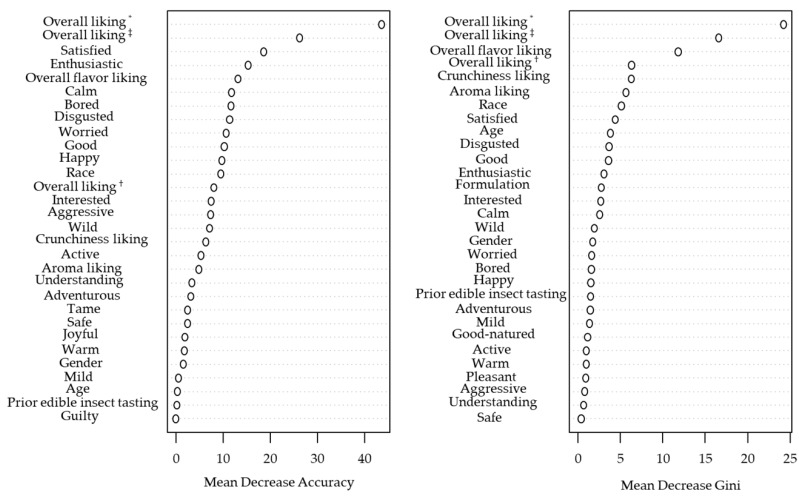
Random forest classifier variables importance plots for after-tasting (after edible cricket protein (ECP) statement) purchase intent (PI) prediction. ^†^ Before-tasting moment; ^‡^ after-tasting (before ECP statement) moment; * after-tasting (after ECP statement) moment. Emotions included in the model were from the after-tasting (after ECP statement) moment.

The holistic approach of incorporating emotions and other variables that may be orthogonal to the product liking dimension has proven to be an efficient tool to provide information beyond liking and to better understand consumers’ behavior [27,42,64]. A random forest (RF) classifier was used in this study to predict the after-tasting (after ECP claim) PI based on demographic variables, formulations, sensory likings, OL from the before and after-tasting (before and after ECP statement) moments, and elicited emotions in the after-tasting (after ECP statement) moment. Figure 9 displays the classifier’s performance in terms of its sensibility and specificity at different classification thresholds. The obtained area under the curve (AUC = 0.91) shows that the model accurately discriminates among consumers willing to purchase and those not willing to purchase the product. Moreover, this model that obtained an out-of-bag accuracy of 84.52% provided input variables’ relative importance for the PI prediction as illustrated in Figure 10. After-tasting (before and after ECP statement) OL, actual overall flavor liking [39,81], positive-valence “satisfied” emotion [26,82], and negative-valence “disgusted” [23,58,83] emotion were common top-10 predictors for the after-tasting informed PI as determined by the mean decrease in classification accuracy and the mean decrease in node impurity when the variable is permuted and split, respectively. In addition, high and low activation/arousal emotions (“enthusiastic,” “calm,” “bored,” and “worried”) [84] and the “good” positive-valence emotion [85] were important inputs to obtain an accurate PI prediction, whereas before-tasting aroma and OL, after-tasting (before ECP statement) crunchiness liking [8], race [44], and age [45,86] were important variables to achieve a higher node purity. According to the RF partial dependence plots (data not shown), the odds of PI = Yes increase for either pita chip formulation when the overall acceptability (regardless of tasting and communication of the ECP statement), flavor, and crunchiness likings increase. In addition, the more elicited the after-tasting (after ECP statement) “disgusted” is and the less elicited the after-tasting (after ECP statement) “interested,” “wild,” “understanding,” “safe,” and “good-natured” are, the lower the probability that either pita chip formulation will be purchased. These results emphasize the importance of yielding an adequate liking profile upon tasting (especially an overall flavor and texture liking) and eliciting pleasant and sensation-seeking emotions but also encourage deeper research to find creative solutions that minimize the disgust sensation that has an overall negative impact from all points of view for novelty products formulated with ECP.

## 4. Study Limitations

This research faced many COVID-19 pandemic restrictions in place at the time of the study that led to limited recruitment of participants (*n* = 84). Hence, no consumer segmentation was performed for the analysis, and the demographic distribution of the subjects is neither balanced nor represents the actual distribution of the US population. Therefore, the findings from this study shall be interpreted with caution, and no inferences should be made on the entire population. For future studies, we recommend testing more seasonings and increasing the sample size, exploring gender and previous edible insect consumption segmentations as these two effects presented a significant interaction with the treatments of this study. In addition, we recommend performing a consumer-based descriptive panel to unearth insights and perceptions that may also affect liking, emotions, and consumption of products formulated with edible cricket protein (ECP). Finally, we would like to emphasize that statistical significance does not always imply practical significance. The former measures the likelihood of a difference (e.g., treatment effect) due to random chance while the latter can tell whether the observed effect is large enough to be “useful” in the reality.

## 5. Conclusions

Although communicating the associated benefits of the consumption of edible cricket protein (ECP) has proven to be beneficial in previous studies, the ECP statement used in this study did not significantly affect the overall liking of the treatments. Similar to the pattern observed for the after-tasting (before ECP statement) OL, after communicating the ECP statement, the plain and Italian treatments presented similar OL ratings, which were significantly higher than for the Cajun treatment. However, tasting and communicating the ECP statement affected the treatments’ emotional profiles more than formulation, which in turn affected the overall product liking in the different moments. This suggests that, although expectations, disconfirmations, and product claims may not have a direct effect on product liking, they may still indirectly affect the overall product acceptability via emotional elicitation. In this study, the plain treatment had both valence and activation/arousal terms discriminate across moments, but only valence and mostly activation/arousal emotions discriminated across moments for the Italian and Cajun treatments, respectively. Minimal differences were observed across the formulations’ emotional profiles; rather larger discrimination was observed across moments within each formulation. This research found that evoking “interested” and “adventurous” emotions plays a significant role as drivers of product liking regardless of the formulation and moment. On the other hand, our results showed that the PI can be improved if the sensory profile of products containing ECP is optimized and through the elicitation of pleasant emotions upon tasting while decreasing the “disgusted” feeling. This research may serve as a guide to optimize novel product development incorporating ECP to foods that are appealing for the market niche they are intended to.

## Figures and Tables

**Table 2:** Demographic profile of participants from the consumer study.

Demographic Variables	Levels	*n*	%
Gender	Female	41	48.81%
	Male	43	51.19%
Age group	18–22	45	53.57%
	23–29	24	28.57%
	30–39	10	11.90%
	40–49	2	2.38%
	50–59	1	1.19%
	≥60	2	2.38%
Race	Asian	5	5.95%
	African American	22	26.19%
	Latino	14	16.67%
	Caucasian	41	48.81%
	Other	2	2.38%
Previously consumed products containing edible insects	Yes	33	39.29%
	No	51	60.71%

**Table 3:** ANOVA ^†^ table for sensory acceptability ^‡^ of treatments ^§^.

Effects	Aroma		Crunchiness		Overall Flavor		Overall Liking *	
	F Value	Pr > F	F Value	Pr > F	F Value	Pr > F	F Value	Pr > F
Gender	1.81	0.18	1.61	0.21	3.30	0.07	1.70	0.20
Age	0.87	0.51	0.26	0.93	0.79	0.56	0.70	0.63
Race	0.39	0.82	0.66	0.62	0.81	0.52	0.60	0.66
Previous edible insect consumption	0.08	0.78	0.07	0.79	0.01	0.91	0.15	0.70
Formulation	31.63	<0.01	11.88	<0.01	12.24	<0.01	16.56	<0.01
Moment	-	-	-	-	-	-	2.00	0.14
Gender * Previous edible insect consumption	0.45	0.51	0.17	0.68	0.02	0.88	0.00	0.99
Gender * Formulation	4.93	0.01	5.10	0.01	3.36	0.04	3.07	0.05
Gender * Moment	-	-	-	-	-	-	0.32	0.73
Previous edible insect consumption * Formulation	14.23	< 0.01	1.98	0.14	0.04	0.96	0.54	0.58
Formulation * Moment	-	-	-	-	-	-	1.37	0.24
Gender * Formulation * Moment	-	-	-	-	-	-	0.29	0.88

^†^ ANOVA = Analysis of variance 2 genders (female and male), 6 age groups (18–22, 23–29, 30–39, 40–49, 50–59, ≥60 years old), 5 races (Asian, African American, Latino, Caucasian, Other), 2 levels of previous edible insect consumption (yes and no), 3 formulations (plain, Italian, and Cajun), and 3 levels of moment (before tasting, after tasting, and after edible cricket (ECP) protein statement). ^‡^ Liking data from *n* = 84 consumers were collected using a 9-point hedonic scale (1 = dislike extremely, 9 = like extremely) and analyzed by a mixed-effects model with panelists as a random effect. ^§^ Treatments are described in Figure 1. * Overall liking determined at three moments (before tasting, after tasting, and after ECP statement).

**Table 4:** Emotional profile ^†^ elicited by the plain treatment ^‡^ across moments.

Emotions	Plain		
	Before Tasting	After Tasting (Before ECP Statement)	After Tasting (After ECP Statement)
Active	3 ^a^	4 ^a^	6 ^a^
Adventurous	38 ^a,(A)^	24 ^b^	31 ^a,b^
Aggressive	0 ^b^	5 ^a^	3 ^a,b,(B)^
Bored	9 ^a^	10 ^a^	12 ^a^
Calm	17 ^a^	13 ^a^	13 ^a^
Disgusted	8 ^a^	14 ^a^	11 ^a^
Enthusiastic	9 ^a^	5 ^a,(B)^	8 ^a,(B)^
Free	2 ^a^	0 ^a^	2 ^a^
Good	9 ^b^	18 ^a^	14 ^a,b^
Good-natured	7 ^a^	6 ^a^	11 ^a^
Guilty	3 ^a^	0 ^a^	0 ^a^
Happy	5 ^a^	5 ^a^	8 ^a^
Interested	46 ^a^	28 ^b^	27 ^b^
Joyful	3 ^a^	1 ^a^	2 ^a^
Loving	0 ^a^	0 ^a^	0 ^a^
Mild	11 ^a(A,B)^	12 ^a^	12 ^a^
Nostalgic	3 ^a^	0 ^a^	0 ^a^
Pleasant	7 ^a^	14 ^a^	10 ^a^
Satisfied	4 ^b^	16 ^a,(A,B)^	17 ^a^
Safe	5 ^a^	5 ^a^	6 ^a^
Tame	5 ^a^	6 ^a^	4 ^a^
Understanding	4 ^b^	7 ^a,b^	12 ^a^
Warm	0 ^a^	1 ^a,(B)^	2 ^a,(B)^
Wild	8 ^a^	5 ^a^	6 ^a^
Worried	14 ^a^	9 ^a^	7 ^a^

^†^ Frequency of emotions for the plain treatment across moments (before tasting, after tasting, and after edible cricket protein (ECP) statement) from *n* = 84 consumers analyzed by two-sided Cochran’s Q test with Marascuilo and McSweeney procedure. Different lowercase/uppercase letters within a row represent significant (*p* < 0.05) differences across moments for the plain treatment/treatments (Table 5 and Table 6) within a given moment. ^‡^ Treatments are described in Figure 1.

**Table 5:** Emotional profile ^†^ elicited by the Italian treatment ^‡^ across moments.

Emotions	Italian		
	Before Tasting	After Tasting (Before ECP Statement)	After Tasting (After ECP Statement)
Active	6 ^a^	9 ^a^	6 ^a^
Adventurous	29 ^a,(B)^	24 ^a^	26 ^a^
Aggressive	2 ^a^	3 ^a^	2 ^a,(B)^
Bored	9 ^a^	6 ^a^	6 ^a^
Calm	14 ^a^	10 ^a^	8 ^a^
Disgusted	10 ^a^	13 ^a^	12 ^a^
Enthusiastic	11 ^a^	11 ^a,(A,B)^	8 ^a,(B)^
Free	5 ^a^	3 ^a^	3 ^a^
Good	12 ^a^	19 ^a^	17 ^a^
Good-natured	6 ^b^	9 ^a,b^	14 ^a^
Guilty	3 ^a^	0 ^a^	0 ^a^
Happy	8 ^a^	11 ^a^	12 ^a^
Interested	37 ^a^	27 ^a^	38 ^a^
Joyful	2 ^a^	3 ^a^	1 ^a^
Loving	0 ^a^	1 ^a^	0 ^a^
Mild	19 ^a,(A)^	13 ^a^	11 ^a^
Nostalgic	2 ^a^	1 ^a^	1 ^a^
Pleasant	5 ^b^	12 ^a^	9 ^a,b^
Satisfied	5 ^b^	17 ^a,(A)^	14 ^a^
Safe	6 ^a^	7 ^a^	5 ^a^
Tame	3 ^a^	3 ^a^	3 ^a^
Understanding	4 ^b^	4 ^b^	10 ^a^
Warm	0 ^a^	2 ^a,(B)^	3 ^a,(A,B)^
Wild	6 ^a^	9 ^a^	6 ^a^
Worried	10 ^a^	6 ^a^	5 ^a^

^†^ Frequency of emotions for the Italian treatment across moments (before tasting, after tasting, and after edible cricket protein (ECP) statement) from *n* = 84 consumers analyzed by two-sided Cochran’s Q test with Marascuilo and McSweeney procedure. Different lowercase/uppercase letters within a row represent significant (*p* < 0.05) differences across moments for the Italian treatment/treatments (Table 4 and Table 6) within a given moment. ^‡^ Treatments are described in Figure 1.

**Table 6:** Emotional profile ^†^ elicited by the Cajun treatment ^‡^ across moments.

Emotions	Cajun		
	Before Tasting	After Tasting (Before ECP Statement)	After Tasting (After ECP Statement)
Active	4 ^a^	5 ^a^	6 ^a^
Adventurous	40 ^a,(A)^	25 ^b^	27 ^b^
Aggressive	1 ^b^	9 ^a^	9 ^a,(A)^
Bored	5 ^a^	8 ^a^	6 ^a^
Calm	11 ^a^	7 ^a^	8 ^a^
Disgusted	14 ^a^	19 ^a^	16 ^a^
Enthusiastic	11 ^a^	13 ^a,(A)^	17 ^a,(A)^
Free	4 ^a^	0 ^a^	2 ^a^
Good	11 ^a^	14 ^a^	12 ^a^
Good-natured	7 ^a^	6 ^a^	8 ^a^
Guilty	2 ^a^	1 ^a^	0 ^a^
Happy	3 ^a^	4 ^a^	5 ^a^
Interested	41 ^a^	27 ^b^	32 ^a,b^
Joyful	4 ^a^	2 ^a^	3 ^a^
Loving	0 ^a^	0 ^a^	0 ^a^
Mild	10 ^a,(B)^	13 ^a^	12 ^a^
Nostalgic	2 ^a^	1 ^a^	1 ^a^
Pleasant	4 ^a^	6 ^a^	5 ^a^
Satisfied	8 ^a^	6 ^a,(B)^	9 ^a^
Safe	7 ^a^	3 ^a^	5 ^a^
Tame	6 ^a^	3 ^a^	2 ^a^
Understanding	5 ^a^	7 ^a^	9 ^a^
Warm	1 ^b^	9 ^a,(A)^	8 ^a,b,(A)^
Wild	7 ^a^	11 ^a^	10 ^a^
Worried	12 ^a^	9 ^a,b^	4 ^b^

^†^ Frequency of emotions for the Cajun treatment across moments (before tasting, after tasting, and after edible cricket protein (ECP) statement) from *n* = 84 consumers analyzed by two-sided Cochran’s Q test with Marascuilo and McSweeney procedure. Different lowercase/uppercase letters within a row represent significant (*p* < 0.05) differences across moments for the Cajun treatment/treatments (Table 4 and Table 5) within a given moment. ^‡^ Treatments are described in Figure 1.

## Data Availability

The data that support the findings of this study are available from the corresponding author upon reasonable request.
